# The Incidence and Characteristics of Oral Candidiasis in Patients Hospitalized for SARS-CoV-2 Infection During the Circulation of Alpha, Beta, and Delta Variants

**DOI:** 10.3390/microorganisms12102090

**Published:** 2024-10-18

**Authors:** Elena Camelia Kouris, Sînziana Irina Mirea, Monica Luminița Luminos, Victor Daniel Miron

**Affiliations:** 1 Carol Davila University of Medicine and Pharmacy, 020021 Bucharest, Romania; 2 National Institute of Infectious Diseases ”Prof. Dr. Matei Balș”, 021105 Bucharest, Romania

**Keywords:** oral candidiasis, COVID-19, SARS-CoV-2, alpha, beta, delta

## Abstract

Background: Oral candidiasis has been documented in patients with SARS-CoV-2 infection, with varying prevalence rates across geographic regions and patient demographics. This study aimed to ascertain the incidence, characteristics, and risk factors associated with the development of oral candidiasis in patients hospitalized for SARS-CoV-2 infection in a tertiary infectious diseases hospital in Romania. Methods: A retrospective analysis was conducted on adult patients hospitalized between March 2020 and December 2022 with moderate or severe forms of SARS-CoV-2 infection, for whom a culture of lingual scrapings for *Candida* spp. was performed. Results: A total of 294 patients were deemed eligible for inclusion in the analysis, with an incidence rate of oral candidiasis of 17.0%. The incidence of oral candidiasis was 4.2 times higher in patients with severe forms of SARS-CoV-2 infection compared to those with moderate forms. Patients with a diagnosis of COVID-19 and oral candidiasis were more likely to receive antibiotics (98.0% vs. 86.1%, *p* = 0.017) and corticosteroids (100% vs. 83.6%, *p* = 0.003) than those without oral candidiasis. These findings were associated with a 19% higher relative risk of developing oral candidiasis for patients who received corticosteroid therapy compared to those who did not, and a 13% higher relative risk for those who were administered antibiotics compared to those who were not. The presence of respiratory insufficiency increased the odds of oral candidiasis association 4.7-fold (88.0% vs. 61.1%, *p* < 0.001). Conclusions: Although the data have been analyzed retrospectively, we have shown that individuals with severe forms of COVID-19 exhibited an elevated risk of developing oral candidiasis. The administration of antibiotics and corticosteroids was identified as a positive predictor for the development of oral candidiasis. The data presented here suggest that a key aspect of the therapeutic management of patients with SARS-CoV-2 infection should include the implementation of preventive measures to minimize the risk of secondary fungal infections.

## 1. Introduction

Severe acute respiratory syndrome coronavirus 2 (SARS-CoV-2) infection has constituted a significant challenge for global health systems, particularly in the context of the pandemic it has precipitated [1,2,3]. The clinical manifestations of SARS-CoV-2 infection range from asymptomatic to severely symptomatic forms of illness, including acute respiratory distress syndrome and multiorgan failure. The most commonly reported complications include pulmonary, cardiovascular, and neurologic impairment, each of which is manifested by a varied spectrum of clinical symptoms, thereby indicating the complexity of SARS-CoV-2 infection [4]. The impact of the SARS-CoV-2 pandemic has been significantly amplified by the emergence and spread of new variants of the virus, in particular the Alpha, Beta, and Delta variants. These variants have demonstrated an increased capacity for transmission in comparison to the wild strain, resulting in the rapid expansion of cases on a global scale and a concomitant increase in pressure on public health systems. Moreover, these variants demonstrated augmented virulence, as evidenced by a greater prevalence of severe disease manifestations and, consequently, an elevated incidence of hospitalization and mortality [5,6].

A considerable number of patients with coronavirus disease 2019 (COVID-19) also presented clinical manifestations of the disease that affected the oral cavity [7]. The oral mucosa, tongue, and salivary glands exhibit a high density of ACE2 receptors, which serve a critical function in facilitating the binding of SARS-CoV-2 [8]. These receptors facilitate the virus’s entry into cells, thereby triggering a spectrum of local manifestations. Furthermore, SARS-CoV-2 infection has the potential to impact the oral microbiota and the natural equilibrium of bacteria and other microorganisms in the oral cavity. The altered microbiota can create an environment favorable for the development of opportunistic infections, such as oral candidiasis or other fungal and bacterial infections [7,9,10]. The most commonly observed changes in the oral cavity during the course of SARS-CoV-2 infection were alterations in taste perception, manifested as dysgeusia or ageusia, the occurrence of oral mucosal lesions, such as vesicular lesions, oral mucosal erosions, and mucositis, changes in salivary gland function, including xerostomia and sialadenitis, bone and periodontal lesions, and the development of secondary lesions, most commonly manifesting as oral candidiasis [9,11].

Viral infections can compromise the host’s immune function, leading to secondary fungal infections. Oral candidiasis is a fungal infection caused by various *Candida* species, mainly due to an imbalance in the oral microbiota [12,13,14]. It has been described in patients with COVID-19 since the beginning of the pandemic [15,16], particularly in those hospitalized and undergoing aggressive treatment. The opportunistic infection is favored by the widespread use of corticosteroids and antibiotics, and by the altered host immunity characteristic of patients with severe forms of SARS-CoV-2 infection. A number of studies have demonstrated that patients with comorbidities and those requiring intensive care are more susceptible to developing oral candidiasis, which further complicates the management of SARS-CoV-2 infection [17,18,19].

The objective of this study was to ascertain the prevalence, characteristics, and risk factors associated with the development of oral candidiasis in patients hospitalized for SARS-CoV-2 infection, manifesting in either moderate or severe forms of the disease.

## 2. Methods

We conducted a retrospective study among patients hospitalized for proven SARS-CoV-2 infection on the 10th ward of the National Institute of Infectious Diseases “Prof. Dr. Matei Balș” (NIID) between March 2020 and December 2022.

This analysis included all adult patients (over 18 years of age) diagnosed with moderate or severe forms of COVID-19 at the time of admission, for whom a tongue scraping culture was performed in order to identify *Candida* spp. and who had specific changes indicative of candidiasis in the oral cavity detected at clinical examination, according to medical records. Patients under the age of 18, those with mild disease, patients for whom no tongue scrape culture was performed, and those with incomplete or inconclusive medical records were excluded.

The identification of eligible patients was performed by a consecutive review of the medical records of all patients hospitalized for COVID-19 on the 10th ward of the National Institute of Infectious Diseases (NIID) during the study period. During this period, the NIID operated as a dedicated hospital for the treatment of patients with confirmed or suspected cases of SARS-CoV-2 infection. Consequently, the medical records of all patients admitted to the hospital during the study period were reviewed and selected according to the predefined inclusion and exclusion criteria.

COVID-19 baseline disease severity is categorized as follows: mild (patients with symptoms but no dyspnea and no pneumonia on chest CT or X-ray), moderate (patients with symptoms and pneumonia detectable on chest CT or X-ray), severe (patients with symptoms and pneumonia requiring oxygen support due to ambient air peripheral oxygen saturation of 93% or lower, or a respiratory rate of 30 or more per minute), and critical (patients needing intensive care for acute respiratory distress, sepsis, altered consciousness, or multiple organ dysfunction due to COVID-19) [20,21]. Critical forms of the disease were not included in the analysis because they were hospitalized in the intensive care unit.

For each patient enrolled in the study, medical records were reviewed and data were extracted on age, sex, associated chronic diseases, the form of disease, the variant of SARS-CoV-2, clinical symptoms at the time of hospitalization, and the results of blood tests on admission, including total leukocyte count, neutrophil count, lymphocyte count, and others. Fibrinogen, C-reactive protein, ferritin, interleukin 1 (IL-1), interleukin 6 (IL-6), lactate dehydrogenase (LDH), alanine aminotransferase (ALT), aspartate aminotransferase (AST), amylase, lipase, the treatment received (antibiotics, corticosteroids, biologic therapy, anticoagulants, antifungal therapy), and the duration of hospitalization were also recorded. Any deviation from the typical laboratory range for blood tests was classified as either decreased or increased in accordance with standard practice.

The data were analyzed using SPSS for Windows, version 25. A chi-square test was employed for the comparative analysis of categorical variables. The frequencies, percentages, and odds ratio (OR) or relative risk (RR) values, as appropriate, are presented together with the confidence interval (95% CI). A comparative analysis of continuous variables was conducted using the Mann–Whitney U test, as the data were not normally distributed. The median and interquartile range (IQR, 25th to 75th percentiles) are presented. A *p*-value of less than 0.05 was considered statistically significant.

## 3. Results

A total of 1179 adult patients were hospitalized during the study period on the ward and were subjected to analysis in our study. Of these, 294 patients, representing 24.9% of the total, were deemed eligible and included in the analysis (Supplementary Material). The median age of all patients included was 52 years (IQR: 43, 64 years), and the female-to-male ratio was 1:1. Of these, 63.9% (n = 188) had severe forms of SARS-CoV-2 infection, the majority of which occurred during the Beta variant circulation period. A total of 50 patients were found to have *Candida* spp., identified in their tongue scraping culture, resulting in an incidence of oral candidiasis of 17.0%. Patients with severe forms of SARS-CoV-2 infection exhibited a 4.2-fold increased risk of developing oral candidiasis compared to those with moderate forms (22.9% vs. 6.6%, OR = 4.2, 95% CI: 1.8–9.7, *p* < 0.001).

The presence of candidiasis was more common among females, and no significant differences in median age were identified between the two groups, although patients in the group with oral candidiasis tended to be older compared to patients in the group without oral candidiasis (55.5 years vs. 51.5 years, *p* = 0.052). The majority of patients (74.1%, n = 218) had at least one chronic disease. The most prevalent conditions in both groups were obesity and hypertension, with no significant intergroup differences (Table 1).

In terms of laboratory parameters, we identified that the median values of inflammatory markers such as C-reactive protein (81.5 mg/dL vs. 70 mg/dL, *p* = 0.026), fibrinogen (519.5 mg/dL vs. 474.5 mg/dL, *p* = 0.048), and ferritin (840.1 ng/dL vs. 540.5 ng/dL, *p* = 0.007) were significantly higher among patients with oral candidiasis compared to those without oral candidiasis (Table 2). No significant differences were identified between the groups with regard to IL-1 or IL-6. However, it is notable that a considerable proportion of patients in the study cohort (73.5%, n = 216) exhibited elevated IL-1 values. A decreased lymphocyte count was the most common modification of blood cell count (77.6%, n = 228), with no statistically significant differences between the two groups (82.0% vs. 76.6%, *p* = 0.408). Additionally, elevated LDH values above the normal range were associated with a 2.5-fold increased risk of oral candidiasis (88.0% vs. 74.6%, OR = 2.5, 95% CI: 1.01–6.1, *p* = 0.040).

Patients with confirmed cases of SARS-CoV-2 infection who also exhibited signs of oral candidiasis received antibiotics (*p* = 0.017) and corticosteroids (*p* = 0.003) significantly more frequently than those who did not present with oral candidiasis. These findings were associated with a 19% higher relative risk of developing oral candidiasis for patients who received corticosteroids compared to those who did not (RR = 1.19, 95% CI: 1.13–1.26) and a 13% higher relative risk for those who took antibiotics compared to those who did not (RR = 1.13, 95% CI: 1.06–1.21). Furthermore, the presence of respiratory failure was associated with an increased odds ratio of 4.7 for the development of oral candidiasis (88.0% vs. 61.1%, OR = 4.7, 95% CI: 1.9–11.4, *p* < 0.001). The median length of hospitalization was 3 days longer for patients with candidiasis than for those without (12.5 days vs. 9.5 days, *p* = 0.001, Table 3). The median duration of hospitalization for the study patients was 10 days (IQR: 7, 14).

## 4. Discussion

In this study, we conducted a retrospective analysis of 294 patients hospitalized for COVID-19 and tested by culture from lingual scraping for the presence of *Candida* spp. The incidence of oral candidiasis in our study was 17%. The results of our study are higher than those reported in previous studies. Di Spirito et al. [22] showed an oral candidiasis rate of 10.74% in a cohort of 4925 adults. Babamahmoodi et al. [23] demonstrated that 2.9% of 4133 patients with SARS-CoV-2 infection developed oral candidiasis. Salehi et al. [24] identified an incidence rate of 5%. The observed variations in incidence may be attributed to several factors related to differences in methodology between studies. These include patient selection and inclusion and/or exclusion criteria. An illustrative example is the study by Salehi et al. [24], which excluded patients with severe forms of acute respiratory distress syndrome. It is also possible that geographic and demographic variations may play an important role. A number of factors, including access to medical care, hospitalization conditions, treatments used, and even individual patient characteristics, may influence the risk of infection with *Candida* spp.

In our study, the occurrence of oral candidiasis was more frequent in females than in males. Furthermore, the median age was 4 years higher among those with oral candidiasis (55.5 years vs. 51.5 years, *p* = 0.0052). Additionally, the majority of patients with oral candidiasis had at least one chronic disease, with the most commonly described conditions being obesity, hypertension, cardiovascular disease, and diabetes mellitus. Similarly, Riad et al. identified, in an analysis of 63 cases of oral candidiasis, a mean age of 59.5 years and a female predominance of 56.7% [25]. Other studies [23,26] have shown that patients with COVID-19 who developed oral candidiasis often had comorbidities such as diabetes, hypertension, and cardiovascular disease. Another study found that patients with COVID-19 and oral candidiasis had a similar mean age in the cases and the control group (61.2 years versus 55.1 years), and comorbidities such as diabetes and cardiovascular disease were more common among those with oral candidiasis [27]. Moreover, a study conducted in a university hospital in a middle-income country revealed that hypertension and diabetes were the most prevalent chronic conditions among critically ill patients with COVID-19 who developed fungal infections, including oral candidiasis [28].

Severe forms of COVID-19 were associated with a higher incidence of oral candidiasis compared with moderate forms of the disease (22.9% vs. 6.6%). This trend was observed in most studies [11,26,27,28]. The mechanisms by which oral candidiasis is encountered in the context of SARS-CoV-2 infection can be both direct and indirect. The development of oral candidiasis has been associated with a number of risk factors, including hospitalization in the intensive care unit, mechanical ventilation of patients, poor oral cavity hygiene, low salivary flow, and background treatment of patients with COVID-19 [29]. Most severe cases of COVID-19 benefit from aggressive treatment, including corticosteroids, antibiotics, and biologic therapy. In our study, although we did not include patients with critical forms of the disease requiring intensive care hospitalization, we had patients with severe forms that required complex therapeutic management. The administration of antibiotics, particularly broad-spectrum antibiotics, is a significant contributing factor in the development of oral candidiasis. *Candida* spp. are essential components of the human microbiota, naturally colonizing the oral, digestive, and vaginal tracts. In these environments, *Candida* spp. coexist in a dynamic equilibrium with other commensal microorganisms, contributing to the maintenance of local homeostasis and mucosal barrier integrity. This balance is regulated through complex mechanisms, including microbial interactions, competition for nutrients, and host immune responses. However, disruptions in this environment, such as antibiotic-induced dysbiosis, immunosuppression, or hormonal changes, can promote the overgrowth of *Candida* spp., leading to pathological conditions like oral or vulvovaginal candidiasis. This symbiotic yet conditionally pathogenic relationship highlights the importance of understanding host–*Candida* interactions for the development of effective prevention and treatment strategies [15,26,30,31]. The use of antibiotic therapy increases the risk of dysmicrobism and favors the multiplication of opportunistic germs such as *Candida* spp. [24,32,33]. Corticosteroid therapy administered for COVID-19 may increase the risk of developing candidiasis in various localizations by decreasing the immune response, favoring the development of dysmicrobism, and transiently increasing serum glucose levels, which creates an environment favorable for the development of candidiasis [34,35,36]. Biologic therapy reduces the immune response and thus may favor the multiplication of different types of *Candida* spp. [37].

In terms of laboratory investigations, a significant association was observed between elevated levels of ferritin, C-reactive protein, and fibrinogen and the development of oral candidiasis in patients with SARS-CoV-2 infection. These elevated laboratory parameters are indicative of an enhanced inflammatory response commonly seen in COVID-19, particularly in its severe and critical forms. Furthermore, patients exhibiting a robust inflammatory response often require complex therapeutic interventions, including prolonged use of immunosuppressive agents, broad-spectrum antibiotics, and corticosteroids. Such treatments can disrupt the normal oral microbial flora and immune defenses, thereby predisposing these patients to the development of opportunistic infections like oral candidiasis. This underscores the importance of closely monitoring inflammatory markers in COVID-19 patients and implementing preventive measures to manage the risk of secondary infections. Similar outcomes have been documented in other studies, wherein an elevated inflammatory biological syndrome was observed. A study conducted in Turkey from 1 March 2020 to 1 April 2021 in a group of 80 patients with systemic candidiasis revealed increased prolactin, ferritin, and leukocytosis among patients with candidemia [38]. In other studies, the majority of patients with oral candidiasis had lymphopenia, which is closely related to the occurrence of fungal infections [39]. These findings were also identified in our case series, in which 82% of patients with oral candidiasis exhibited a low lymphocyte count.

The treatment regimen prescribed for patients with SARS-CoV-2 infection and concomitant oral candidiasis was similarly complex. All patients received corticosteroid therapy (100%), and the majority of them also received antibiotics and/or biological therapy. As observed in our study and in other studies, an association between severe forms requiring aggressive treatment and the occurrence of *Candida* spp. infections has been documented. In a study conducted in Iran, 89% of patients received corticosteroid therapy and 55% received broad-spectrum antibiotic therapy [23]. In a separate study conducted in China prior to the pandemic, the correlation between corticosteroid therapy and the emergence of non-albicans *Candida* spp. infections was identified [40]. Concurrently, the administration of oxygen to patients with acute respiratory failure has been observed to increase the risk of developing oral candidiasis. This is due to the reduction in salivary secretion and subsequent drying of the oral mucosa, which occurs as a result of the oxygen therapy. In addition, critically ill patients who required oxygen therapy exhibited inadequate daily oral hygiene practices. These factors collectively resulted in significant local imbalances, leading to the proliferation of *Candida* spp [26,30]. In our analysis, it was observed that 90% of patients diagnosed with oral candidiasis were administered systemic antifungal therapy. Effective management of oral candidiasis necessitates an integrative approach that combines the use of antifungal agents with strict adherence to oral hygiene protocols. For cases presenting with mild symptoms, topical antifungal treatment is typically adequate. However, systemic therapy is generally reserved for patients with severe manifestations or those with conditions unresponsive to topical treatment [41]. Additionally, it is crucial to counsel patients on mitigating risk factors to prevent recurrence. This includes managing underlying conditions such as diabetes, encouraging smoking cessation, and promoting nutritional improvements. These measures play a vital role in the holistic management of oral candidiasis and in reducing the likelihood of recurrent infections [41].

Our study has a number of limitations that deserve to be recognized and discussed. Firstly, the retrospective nature of the data used and the patient selection restricted to a single hospital ward impose certain constraints on the generalizability of the results. Secondly, this study did not include data on the microbiological and resistance characteristics of *Candida* spp. Additionally, a short- and medium-term follow-up of patients, which could have provided further insight into the potential recurrence of oral candidiasis, was not conducted. However, it is important to emphasize that we were able to include a significant number of patients, generating an extensive dataset. This allowed us to identify relevant characteristics and risk factors for the development of oral candidiasis among Romanian patients hospitalized in the largest infectious diseases hospital in the country and the main coordinating center of the COVID-19 pandemic. Thus, although this study has its limitations, its contributions are essential for the understanding and management of oral candidiasis in the context of SARS-CoV-2 infection.

## 5. Conclusions

Our study revealed a 17.0% incidence rate of oral candidiasis among hospitalized patients with SARS-CoV-2 infection during the circulation of Alpha, Beta, and Delta variants. It was determined that patients with severe forms of the disease were at an elevated risk of developing oral candidiasis. The administration of antibiotics and corticosteroids was identified as a positive predictor for the development of oral candidiasis. These data indicate that the therapeutic management of patients with SARS-CoV-2 infection should include preventive measures to minimize the risk of secondary fungal infections. In light of these findings, it is imperative to conduct a thorough assessment of the necessity of the use of antibiotics and corticosteroids in order to reduce the incidence of oral candidiasis and to enhance the prognosis and quality of life of these patients.

## Figures and Tables

**Table 1:** The general characteristics of the patients included in this study.

Characteristics	All Patients	Group with Oral Candidiasis	Group without Oral Candidiasis	*p*-Value
	N = 294	N = 50	N = 244	
Demographic data				
Female	147 (50.0%)	31 (62.0%)	116 (47.5%)	0.062
Age, years, median (IQR)	52 (43, 64)	55.5 (47, 69)	51.5 (42, 63)	0.052
SARS-CoV-2 variant				
Alpha	52 (17.7%)	4 (8.0%)	48 (19.7%)	0.143
Beta	157 (53.4%)	30 (60.0%)	127 (52.0%)	
Delta	85 (28.9%)	16 (32.0%)	69 (28.3%)	
**Disease form**				
Moderate	106 (36.1%)	7 (14.0%)	99 (40.6%)	<0.001
Severe	188 (63.9%)	43 (86.0%)	145 (59.4%)	
**Chronic condition**				
At least one chronic condition	218 (74.1%)	40 (80.0%)	178 (73.0%)	0.300
Obesity	158 (53.7%)	30 (60.0%)	128 (52.5%)	0.330
High blood pressure	146 (49.7%)	30 (60.0%)	116 (47.5%)	0.108
Diabetes mellitus	57 (19.4%)	12 (24.0%)	45 (18.4%)	0.365
Cardiovascular disease	108 (36.7%)	19 (38.0%)	89 (36.5%)	0.839
Chronic lung disease	17 (5.8%)	4 (8.0%)	13 (5.3%)	0.461
Chronic kidney disease	14 (4.8%)	3 (6.0%)	11 (4.5%)	0.652
Neurological diseases	15 (5.1%)	3 (6.0%)	12 (4.9%)	0.751

IQR—interquartile range.

**Table 2:** Modifications in the laboratory parameters of the patients included in this study.

Feature	All Patients	Group with Oral Candidiasis	Group without Oral Candidiasis	*p*-Value
	N = 294	N = 50	N = 244	
Neutrophil count, cells/µL, median (IQR)	4100 (2800, 6425)	4950 (3125, 7470)	3970 (2705, 6375)	0.069
Increased neutrophil count, n (%)	67 (22.8%)	16 (32.0%)	51 (20.9%)	0.088
Decreased neutrophil count, n (%)	13 (4.4%)	0 (0.0%)	13 (5.3%)	NA
Lymphocyte count, cells/µL, median (IQR)	900 (615, 1200)	720 (600, 1100)	900 (640, 1200)	0.168
Increased lymphocyte count, n (%)	5 (1.7%)	2 (4.0%)	3 (1.2%)	0.167
Decreased lymphocyte count, n (%)	228 (77.6%)	41 (82.0%)	187 (76.6%)	0.408
Fibrinogen, mg/dL, median (IQR)	485 (387, 577.3)	519.5 (447.5, 607.5)	474.5 (370, 560.8)	0.048
Increased fibrinogen, n (%)	218 (74.1%)	42 (84.0%)	176 (72.1%)	0.081
C-reactive protein, mg/L, median (IQR)	71 (30.8, 110)	81.5 (44.5, 143.3)	70 (28, 107.5)	0.026
Increased C-reactive protein, n (%)	284 (96.6%)	50 (100%)	234 (95.9%)	0.145
IL-1, pg/L, median (IQR)	2.4 (0.2, 7.0)	2.8 (0.5, 10.3)	2.4 (0.2, 6.3)	0.460
Increased IL-1, n (%)	91 (31.0%)	17 (34.0%)	74 (30.3%)	0.609
IL-6, pg/L, median (IQR)	59.5 (15.8, 220)	77.5 (10.9, 709.1)	53.5 (16, 182)	0.189
Increased IL-6, n (%)	216 (73.5%)	37 (74.0%)	179 (73.4%)	0.926
LDH, U/L, median (IQR)	315 (250.8, 400.5)	375 (311.8, 486.8)	306 (245.3, 381.5)	<0.001
Increased LDH, n (%)	226 (76.9%)	44 (88.0%)	182 (74.6%)	0.040
Ferritin, ng/mL, median (IQR)	563 (263.3, 1267)	841 (463.3, 1650)	540.5 (240.3, 1212.8)	0.007
Increased ferritin, n (%)	205 (69.7%)	43 (86.0%)	162 (66.4%)	0.006
AST, U/L, median (IQR)	54 (35, 79)	60.5 (39.8, 83)	53.5 (34, 76)	0.130
Increased AST, n (%)	137 (46.6%)	27 (54.0%)	110 (45.1%)	0.249
ALT, U/L, median (IQR)	76 (40.8, 124.5)	80 (58.8, 140.3)	76 (38, 120)	0.224
Increased ALT, n (%)	178 (60.5%)	36 (73.5%)	142 (58.7%)	0.053
Amylase, U/L, median (IQR)	67.5 (48.8, 99)	72.5 (51.8, 99)	66 (48, 99)	0.839
Increased amylase, n (%)	65 (22.1%)	10 (20.0%)	55 (22.5%)	0.693
Lipase, U/L, median (IQR)	167.5 (106.8, 298.5)	175.5 (100.8, 319)	166.5 (109.3, 290.5)	0.626
Increased lipase, n (%)	74 (25.2%)	15 (30.0%)	59 (24.2%)	0.388

LDH—lactate dehydrogenase, ALT—alanine aminotransferase, AST—aspartate aminotransferase, IL-1—interleukin 1, IL-6—interleukin 6, NA—not applicable, and IQR—interquartile range. All units of measurement for blood tests are reported in accordance with the standards set forth by the hospital laboratory (U—unit, L—liter, dL—deciliter, µL—microliter, mg—milligram, pg—picogram, ng—nanogram).

**Table 3:** Management and evolution of cases.

Feature	All Patients	Group with Oral Candidiasis	Group without Oral Candidiasis	*p*-Value
	N = 294	N = 50	N = 244	
Treatment				
Antibiotics	259 (88.1%)	49 (98.0%)	210 (86.1%)	0.017
Biological therapy	139 (47.3%)	27 (54.0%)	112 (45.9%)	0.239
Corticosteroids	254 (86.4%)	50 (100%)	204 (83.6%)	0.003
Anticoagulants	292 (99.3%)	50 (100%)	242 (99.2%)	0.521
Antifungal therapy	70 (23.8%)	45 (90.0%)	25 (10.2%)	<0.001
Evolution				
Days since onset, median (IQR)	7 (5, 10)	7 (6, 10)	7 (5, 9)	0.219
Duration of hospitalization, days, median (IQR)	10 (7, 14)	12.5 (9, 18.25)	9.5 (7, 13.75)	0.001
Respiratory failure, n (%)	193 (65.6%)	44 (88.0%)	149 (61.1%)	<0.001

IQR—interquartile range.

## Data Availability

The data are available upon reasonable request from the corresponding author.

## Supplementary Materials

The following supporting information can be downloaded at: https://www.mdpi.com/article/10.3390/microorganisms12102090/s1, Study flow chart.
