# Supplementary figures and images for: The Incidence and Characteristics of Oral Candidiasis in Patients Hospitalized for SARS-CoV-2 Infection During the Circulation of Alpha, Beta, and Delta Variants
Source: Microorganisms. 2024 Oct 18;12(10):2090. doi: 10.3390/microorganisms12102090 (PMC11509929; doi:10.3390/microorganisms12102090)

**Supplementary material. Study flow chart**

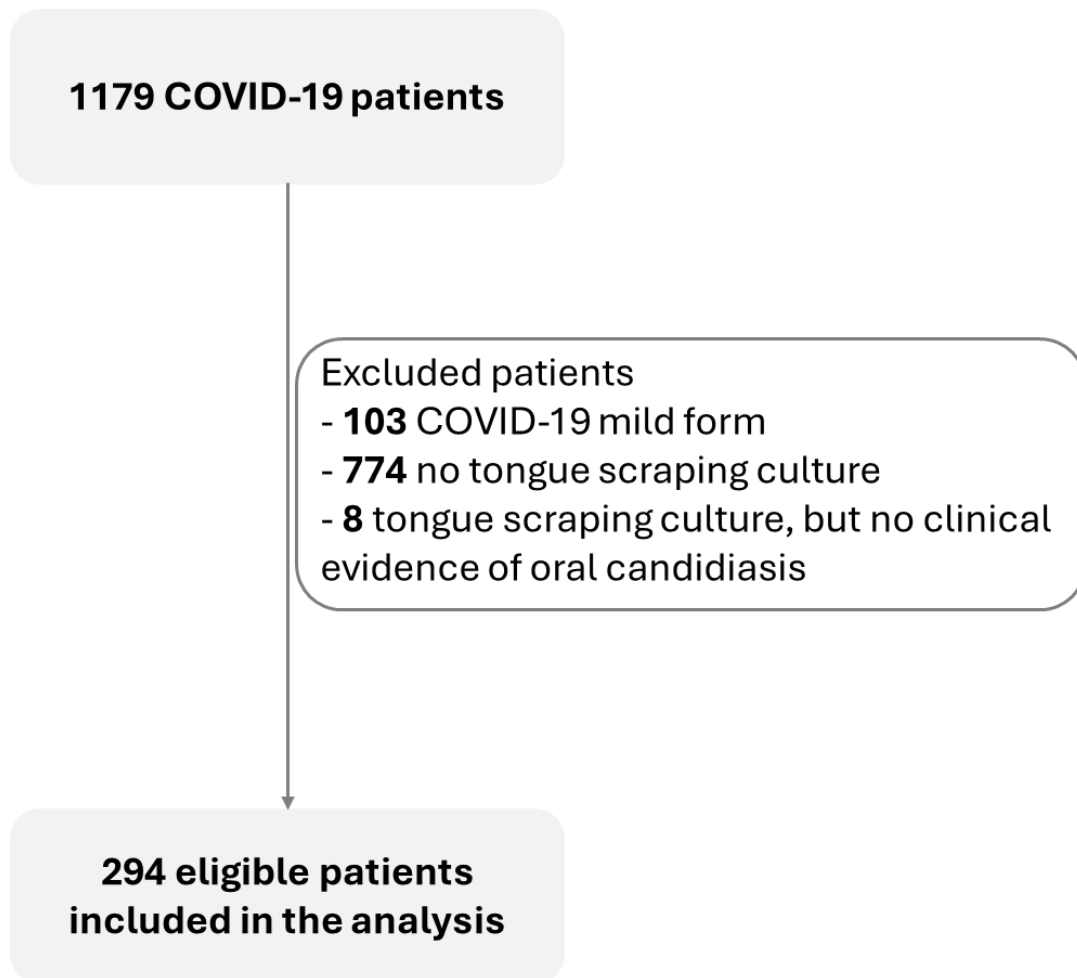

Supplement: Supplementary file 1 [file microorganisms-12-02090-s001.zip › microorganisms-3152575-supplementary.pdf]
